# Projecting the Pandemic Trajectory through Modeling the Transmission Dynamics of COVID-19

**DOI:** 10.3390/ijerph19084541

**Published:** 2022-04-09

**Authors:** Vahideh Vakil, Wade Trappe

**Affiliations:** Department of Electrical and Computer Engineering, Rutgers, The State University of New Jersey, Piscataway, NJ 08854, USA

**Keywords:** COVID-19, vaccination, transmission dynamics, epidemiology, compartmental modeling, SIRS

## Abstract

The course of the COVID-19 pandemic has given rise to many disease trends at various population scales, ranging from local to global. Understanding these trends and the epidemiological phenomena that lead to the changing dynamics associated with disease progression is critical for public health officials and the global community to rein in further spread of this and other virulent diseases. Classic epidemiological modeling based on dynamical systems are powerful tools used for modeling and understanding diseases, but often necessitate modifications to the classic compartmental models to reflect empirical observations. In this paper, we present a collection of extensions to the classic SIRS model to support public health decisions associated with viral pandemics. Specifically, we present models that reflect different levels of disease severity among infected individuals, capture the effect of vaccination on different population groups, capture the effect of different vaccines with different levels of effectiveness, and model the impact of a vaccine with varying number of doses. Further, our mathematical models support the investigation of a pandemic’s trend under the emergence of new variants and the associated reduction in vaccine effectiveness. Our models are supported through numerical simulations, which we use to illustrate phenomena that have been observed in the COVID-19 pandemic. Our findings also confirm observations that the mild infectious group accounts for the majority of infected individuals, and that prompt immunization results in weaker pandemic waves across all levels of infection as well as a lower number of disease-caused deaths. Finally, using our models, we demonstrate that, when dealing with a single variant and having access to a highly effective vaccine, a three-dose vaccine has a strong ability to reduce the infectious population. However, when a new variant with higher transmissibility and lower vaccine efficiency emerges, it becomes the dominant circulating variant, as was observed in the recent emergence of the Omicron variant.

## 1. Introduction

The COVID-19 pandemic has brought into focus the importance of being able to predict the trajectory of viral pandemics, and how such forecasts can be valuable in guiding public health policies. Over the course of the past few years, the world has grappled with numerous challenges associated with the SARS-CoV-2 coronavirus (i.e., COVID-19), and looking back reveals a plethora of unfortunate scenarios where public health officials struggled with difficult challenges, ranging from the lack of sufficient resources in public hospital systems, to the importance of testing kits for detecting and identifying patients and “carriers” of COVID-19, and to the emergence of many different variants of the original COVID-19 virus. This latter scenario is perhaps one of the most disconcerting, as it causes public health officials to worry whether the waves of COVID-19 will continue in perpetuity, potentially requiring significant investment in public health infrastructures, as the SARS-CoV-2 virus has evolved through numerous mutational variants that have demonstrated more potent disease pathology. It further highlights the importance of being able to create forecasts that go beyond a single disease variant, but that can capture the dynamics and competition between variants.

After two years following the onset of the pandemic, the global health community has been able to amass significant data regarding the impact that COVID-19 has had locally and globally on public health. There are now numerous reports and articles analyzing the pandemic trends, which have explored different approaches to understand the pandemic’s trajectory. Mathematical modeling has been quite useful in illustrating what the world has experienced over the past two years and predicting what the future of the pandemic might look like.

In this paper, we first explore various aspects of the pandemic that have been analyzed and interpreted using COVID-19 transmission dynamics modeling. This includes models that study the disease’s severity level, explore the emergence of new variants, investigate the impact of vaccination strategies, and those that explore the impact of different social behaviors, such as social distancing and lockdown, on the pandemic trends [1,2,3,4,5,6,7,8,9,10,11,12].

Afterwards, we use the epidemiology of COVID-19 to develop a collection of compartmental mathematical models that illustrate the pandemic’s trend and anticipate its trajectory for different situations. The models we present can be helpful in illustrating potential disease trajectories that might arise in different scenarios and to predict the pandemic’s trend based on the status of vaccination efforts and the emergence of variants of concern.

The remainder of the paper is organized as follows. In Section 2, we review published articles that extend classical compartmental models for COVID-19, and the various approaches that have been employed. In Section 3, we revisit the classic susceptible-infectious-recovered (SIR) and susceptible-infectious-recovered-susceptible (SIRS) models to establish a starting point for our work, and examine the effectiveness of these simple models in making predictions for the COVID-19 pandemic. Then, in Section 4, we present our first extended SIRS model, where we have modified SIRS to capture the observation that viral pandemics can demonstrate varying levels of disease infection severity levels. We extend our model in Section 5 to include the impact that vaccination can have on disease progression through a population. Then, in Section 6, we present a model that examines the impact that having a variety of vaccines can have on the population, and use it to explore issues surrounding vaccine efficacy, and complexities associated with distribution of a vaccine. In Section 7, we present a 3-dose vaccine model with separate compartments for vaccinated and unvaccinated individuals. Using this model, we illustrate how it is possible to capture the observed waning in vaccine protection, and the benefits associated with sustaining vaccination and vaccine boosting efforts to prevent resurgence in the spread of the disease. We next extend our models in Section 8 to account for the emergence of new variants. Each of the Section 3, Section 4, Section 5, Section 6, Section 7 and Section 8 concludes with simulation results and a discussion about how the results explain an observed aspect of the pandemic. Finally, we conclude the paper in Section 9.

## 2. Transmission Dynamics and the Pandemic’s Trajectory

In this section, we survey a collection of papers that provide extensions to classic compartmental models and explore various aspects of the COVID-19 pandemic to anticipate the disease’s trajectory. The papers in this section were chosen for their similarities in using epidemiological modeling to investigate pandemic trends, as well as for their differences in the specific aspects of the pandemic they studied.

The authors in [1] estimate the clinical severity and overall symptomatic case fatality risk of COVID-19 from its transmission dynamics using public and published data. They modify the SIR model to simulate the COVID-19 epidemic in Wuhan using several assumptions, including the population’s mobility, the probability density functions associated with the virus incubation period and the time between onset and death in severe cases, and the probability of symptomatic infections. The basic reproduction number, mean serial interval, initial doubling time, intervention effectiveness, and mean time from onset to death are then estimated based on their model.

In [2], the authors modify the susceptible-exposed-infectious-recovered (SEIR) model by adding compartments for asymptomatic and pre-symptomatic individuals. In their model, a fraction of the newly infected patients remain asymptomatic, while the remaining become pre-symptomatic before moving to the symptomatic stage, where they may die from the disease. The remainder of the people recover and are considered to be immune. According to [2], selection favors higher transmission and a shorter time between exposure and infectiousness. The authors also claim that selection favors mutations that put people in the infectious compartment for extended periods of time. They also argue that selection favors a decline in the number of asymptomatic individuals as long as pre-symptomatic and symptomatic individuals remain the primary sources of new infections.

The authors in [3] extend the SEIR model by adding the effect of vaccinations and emergence of new variants. They change transition rates between states associated with infection, viral incubation, recovery, and mortality in their proposed model to incorporate vaccination and variants of concern into the transmission model. The paper classifies the various ways in which models provide valuable information. Calculating the herd immunity threshold and evaluating its limitations, confirming that vaccines can prevent severe infection and transmission but may be less effective against new variants of concern, determining optimal vaccination strategies, and determining that new variants might be more transmissible and lethal than previously circulating strains, are a few of these methods. They also address how models can help in anticipating and preparing for future COVID-19 epidemiological stages.

To capture the durability of immunity, the authors in [4] offer a SIRS-based model with different categories of primary infected and secondary infected individuals, as well as fully susceptible individuals and secondary susceptible individuals. They also include a vaccinated cohort from both primary and secondary susceptible individuals in their extended model. Using these models, they predict the future of COVID-19 cases given different assumptions, such as the impact of vaccination and interventions, e.g., school closures and lockdowns that are reflected in the transition rates between different compartments. They predict complex dynamics for the future of COVID-19 due to the interactions among vaccination effects, reinfection cases, and non-pharmaceutical interventions. The authors in [5] then extend the model proposed in [4] by adding compartments for two-dose vaccinated individuals and capturing the waning immunity of vaccinated people after each dose of a vaccine. They show that a single dose vaccination reduces infections in the short term, but that longer-term effects are dependent on the relative robustness of the immune response induced by a single dosage versus natural and two-dose immunity. They investigate three different selection scenarios and show that a one-dose policy may increase the possibility for antigenic evolution under specific partial population immunity conditions. They emphasize the importance of testing viral loads and quantifying immune responses after a single vaccine dosage, as well as increasing global vaccination efforts.

Another SEIR-based model extension is presented in [6] in which the authors include vaccination, social distancing metrics, and the testing of susceptible individuals in the model. Separate compartments for symptomatic and asymptomatic infectious individuals, as well as an additional compartment for isolated individuals, are included in their model. Then, under the assumption of the deployment of a mass vaccination program, the impacts of uncertainty about the application of social distance actions and testing of susceptible individuals on disease transmission are measured.

The authors in [7] simulate COVID-19 transmission in the United Kingdom using an age-structured SEIR transmission model. They assume gamma distribution for both latent and infectious periods and adjust their model by splitting the exposed and infectious compartments for each age group into multiple subcompartments. They simulate a variety of non-pharmaceutical intervention strategies that include social distancing for various age groups. They confirm that SARS-CoV-2 transmission can be suppressed over a period of months with reasonable levels of social distancing, which is consistent with reported trends. According to their model, social distancing should initially reduce transmission rates to a small range, and the extent of social distancing must be adaptive over time to adjust for susceptible reduction. In a similar modeling approach, the authors in [8] use a deterministic age-structured SEIR model with three age groups to estimate key model parameters and project the number of COVID-19 hospitalizations, using data from Colorado, USA. The likelihood that an infected person develops symptoms and requires hospitalization or ICU care is assumed to be age dependent; however, all individuals, regardless of age, have an equal chance of exposure and infection. Three types of transmission-reducing parameters are involved in the model including social distancing, mask wearing, and symptomatic person self-isolation.

In [9], the authors propose an extension on the SEIR model, which is made up of eight compartments of susceptible, unsusceptible, exposed, infected, hospitalized, critical, dead, and recovered individuals. Due to the social distancing factor, the model assumes that a susceptible person might either become unsusceptible or exposed to the virus. After an incubation period, individuals who have been exposed become infectious. Infectious individuals can remain infected for some period of time and either recover or be hospitalized. People who are hospitalized for some period of time might either recover or become critically ill, necessitating admission to an intensive care unit, and then the patient might either return to the hospital or die. Individuals who have recovered can be infected or have been in the hospital. The simulation results of the model confirm the necessity of the personal protection measures in pandemic containment and suggest an adaptive social distancing approach.

The authors in [10] modify a SEIR-based model to monitor various intervention measures in Uganda. Their model implies that after a latent period, a fraction of those who have been exposed become infectious while still being isolated in institutional quarantines. A fraction of the remaining untraced exposed people become asymptomatic, while the rest become symptomatically infectious. The infectious individuals who stay in the community are then identified and admitted to the hospital. The disease can be lethal, and symptomatic persons may die to it before or after being admitted to the hospital. Individuals who are hospitalized may recover and are considered to be temporary immune to the disease. Their findings reveal that improving contact tracing can only affect the peak and timing of a secondary wave, but cannot eliminate it entirely.

In another example of modifying classic models, the authors of [11] provide a compartmental model of COVID-19 spread in Wuhan, with a focus on super-spreader transmissibility. The model includes compartments for individuals who are susceptible, exposed, symptomatic infectious, super-spreader, asymptomatic infectious, hospitalized, recovered, as well as a compartment for death cases. They provide an approximation for the number of cases and basic reproduction number in the Wuhan outbreak and predict a decline in the daily number of confirmed cases of the disease using this model.

In [12], the authors use a SEIR-based model to examine the various scenarios for the COVID-19 pandemic during a phase involving the easing of restrictions. Their model includes a parameter that simulates the restrictions. Their simulation results suggest substantial spikes of infections as a result of loosening limitations, indicating a subsequent reacceleration of the disease’s spread. They also argue that such undesirable situations could be prevented via a control method of sequential partial lifting limitations.

In a different approach, the authors in [13] use an agent-based model of COVID-19 to study the pandemic in New York state, USA, and the United Kingdom. Unlike compartmental models, in agent-based models an agent’s behavior is determined individually, and their joint behavior is described through the interaction of multiple agents. Given that epidemiological parameters, such as virus transmission rate, initial number of infected people, and the probability of being tested are affected by the demographic and geographical characteristics of a region, the paper calibrates these parameters using COVID-19 data from 2020, for each region. The presented model predicts COVID-19 spread in New York state and the United Kingdom for March 2021, demonstrating that if testing and containment measures were maintained, the number of positive cases in New York State would remain stable while in the UK it would reduce. We note that computational and simulation methods, such as agent-based modeling, allow for health experts to further refine and explore the impact of factors, such as geography and urban/rural differences upon disease spread.

In this paper, we present multiple extensions to the SIRS epidemiological model that can be used to investigate COVID-19’s trajectory, and which allow an epidemiologist to examine varying aspects related to a disease’s trajectory. Unlike the above-mentioned papers, which present a single model and approach, we start from a baseline mathematical model and demonstrate how one can introduce different compartments and population flows within the model to examine aspects of the pandemic’s behavior. We present five different models in this paper and employ these models to investigate various questions about the pandemic’s trajectory:

- In the first extension, we present a model with three different infectious compartments, each representing a different severity level for the disease. Using empirical data associated with the early phase of the COVID-19 pandemic, in which patients experiencing milder disease recovered from infection quicker than more severe cases, we show through simulations that most infections will be mild, although mildly infected individuals retain less durable resistance post-recovery. The recovered patients in this model and all of our other extensions are assumed to return to the susceptible compartment, in contrast to [2], which assumes recovered individuals remain immune.
- In our second extension, we build upon the first model to arrive at a model that includes multiple compartments for varying levels of disease severity, as well as a protected compartment to represent individuals who have been vaccinated. We assume different vaccine effectiveness across different disease severity levels. Further, we include a time-dependent vaccination rate in our model to account for the effect of vaccination initiation time on the pandemic trajectory. Our model and results support the observation that early vaccination reduces the severity of pandemic waves across all severity levels of infection.
- The third extension we present models a vaccination strategy that uses multiple vaccines with varying availability and immunization efficacy, and shows through simulations that vaccine availability and distribution is a major contributor to the timing and peak of the second wave and subsequent waves of the pandemic. This model offers the epidemiologist a tool to explore the impact that additional investments in vaccine production and distribution can have on the progression of the disease through the population.
- The fourth extension includes separate compartments for vaccinated and unvaccinated individuals and is designed to reflect immunization with multiple vaccine doses. This model allows one to explore scenarios in which the population receives varying amounts of vaccine doses, such as occurred with the Pfizer and Moderna mRNA vaccines. The concept of having multiple compartments for different susceptible groups based on their immunization status is similar to [4,5]. However, unlike these papers, we assume direct interactions between the compartments that represent different levels of vaccination as well as susceptible vaccinated and susceptible unvaccinated groups. Further, we demonstrate how the model can be adapted to involve time-dependent vaccination rates for individuals receiving varying quantities of doses. In particular, the model can be used to explore the trade-offs between a vaccination strategy that focuses on administering the first dose to as many people as possible in comparison with progressing individuals to second and third (boosted) doses of the vaccine.
- Finally, our fifth model extension accounts for the emergence of new variants with different levels of transmissibility and vaccine effectiveness. We utilize recent data for the Delta and Omicron variants of COVID-19, and show through simulations that the more transmissible and less-severe Omicron variant, which provides cross-reactive humoral immunity to the Delta variant, will ultimately become the dominant variant. This model provides a mathematical tool for the public health community to explore the dynamics that might arise in the competition between variants.

In the following sections, we first review the classic SIR and SIRS models and examine how they estimate COVID-19’s trends; afterwards, we present our collection of SIRS extensions, which facilitate investigating the COVID-19 trajectory across different aspects of the disease. These factors include the severity of the disease, the effectiveness of multi-dose immunization, and the impact of emerging new variants, but they exclude age and social distancing policies. We note that our model can be extended to include specific subpopulations, such as the elderly or those with pre-existing conditions, by introducing a separate population subcompartment for that particular demographic.

## 3. Compartmental Epidemiological Models for the COVID-19 Pandemic

**Figure 1:**
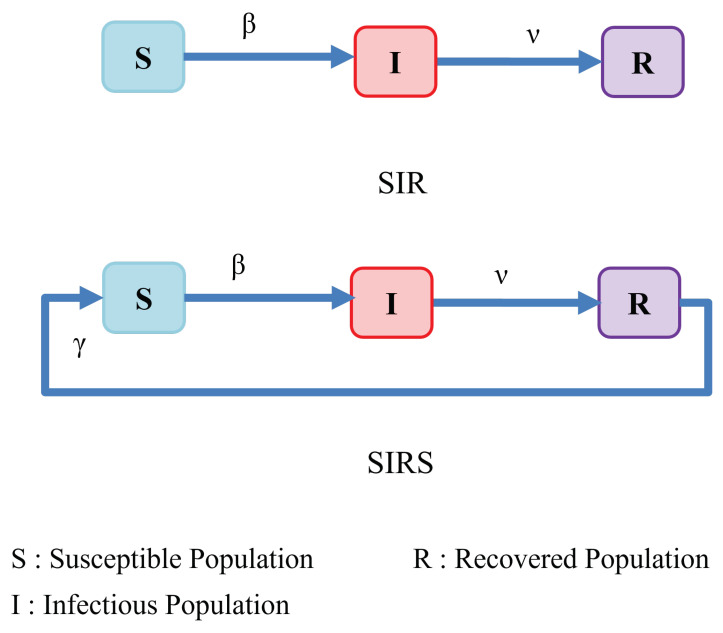
Classic SIR and SIRS models.

A series of papers by Kermack and McKendrick [14,15,16] introduce the basic compartmental models for describing the transmission of communicable diseases. The SIR model, displayed in Figure 1, is a special case of the model proposed by Kermack and McKendrick in 1927, which is the starting point for many epidemic models [17]. This is a compartmental model that is based on simple assumptions about the rates of flow between distinct classes of population members. The population is split into three groups: *S*, *I*, and *R*. S(t) represents the number of people who are susceptible to the disease, or are not affected yet at time *t*. I(t) denotes the number of infected individuals who are presumed to be infectious and capable of spreading the disease to others through contact. R(t) denotes the number of people who have been infected, recovered and, thus, are no longer at risk of becoming infected or spreading infection. SIR is a term used to represent a disease for which infection leads an infected individual to develop (permanent) immunity against reinfection (for example, it is widely recognized that infection with the wild type measles virus provides lifelong immunity). Hence, the model represents a disease with dynamics where individuals transition from the susceptible class *S* to the infectious class *I* with the rate of β, and then to the removed class *R* with the rate of ν, never to be infected again. The dynamics of the system in this model can be expressed by

(1) $$\begin{array}{cc} \frac{dS}{dt} & =-\beta IS,\\ \frac{dI}{dt} & =\beta IS-\nu I,\\ \frac{dR}{dt} & =\nu I, \end{array}$$

where it is assumed that the total population size is constant. When the number of infected individuals increases, i.e., dI/dt>0, the disease spreads and an epidemic begins. Assuming that set of equations (1) is provided for the fraction of the population in each compartment (i.e., normalized by the total population), and that almost everyone is susceptible at the onset of an epidemic ($S_{0}\approx 1$), this implies that $R_{0}=\beta /\nu >1$. $R_{0}$ is called the basic reproduction number and is defined as the expected number of secondary cases produced by a single infection in a fully susceptible population.

However, in many viral infections, the permanent immunity assumed in the SIR model is not always accurate for a variety of reasons, including virus mutation and waning immunity. Therefore, in this paper, we adopt the SIRS model as our starting point, shown in Figure 1, which reflects the epidemiology of incomplete immunization to viral diseases. In this case, previously infected and recovered individuals eventually return to the susceptible compartment after a period of time. The rate of this transition (γ) is defined by the inverse of duration of immunity developed after the prior infection. The following system of equations represents the dynamics of the SIRS model:

(2) $$\begin{array}{cc} \frac{dS}{dt} & =-\beta IS+\gamma R,\\ \frac{dI}{dt} & =\beta IS-\nu I,\\ \frac{dR}{dt} & =\nu I-\gamma R. \end{array}$$

Table 1 contains the description of all rate parameters used throughout the paper. When a model has more than one compartment of each type due to different levels of disease, multiple vaccines, or multiple variants, the corresponding rates will appear with proper indices.

### Simulation Results and Discussion

In this section, we provide simulation results for the classic SIR and SIRS models in which the underlying rate parameters have been tailored to match the early phase of the COVID-19 pandemic (when there was a single variant). We assume a constant population of $N=10^{6}$ with 1 infectious individual. Our simulations are performed in terms of compartments that are represented using fractional populations that have been normalized by *N*. In other words, the initial value for the fraction individuals that are infectious is $I_{0}=1\times 10^{-6}$. Throughout the remainder of this paper, we shall refer to the fraction of infectious population as the normalized size of an infectious compartment *I*. We also assume that the remainder of the population is totally susceptible; thus, the initial value for the fractional susceptible is $S_{0}\approx 1$. We assume the mean value for the infectious period as 1/ν=5 days [4,5,18], with peak infectiousness varying between 3 and 7 days [19]. Unless otherwise specified, we take $R_{0}=3.32$ as the mean value for reproduction number for the original COVID-19 strain, and which is consistent with the herd immunity target of 70% associated with the initial variant. The duration of natural immunity (1/γ) has been assumed in the range of 3 months to 2 years in [4,5]. Moreover, according to a recently published study [20], 1 year after infection, 87% of patients with the wild type variant were still 50% protected against severe disease, while 17% are still protected against detectable disease. However, the same study found that the immunity period against variants B.1.1.7 and B.1.351 was shortened. This trend of reduced immunity to new variants has continued as the Delta and Omicron variants, revealing that viral evolution can lead to significantly reduced levels of acquired protection to emerging variants.

**Figure 2:**
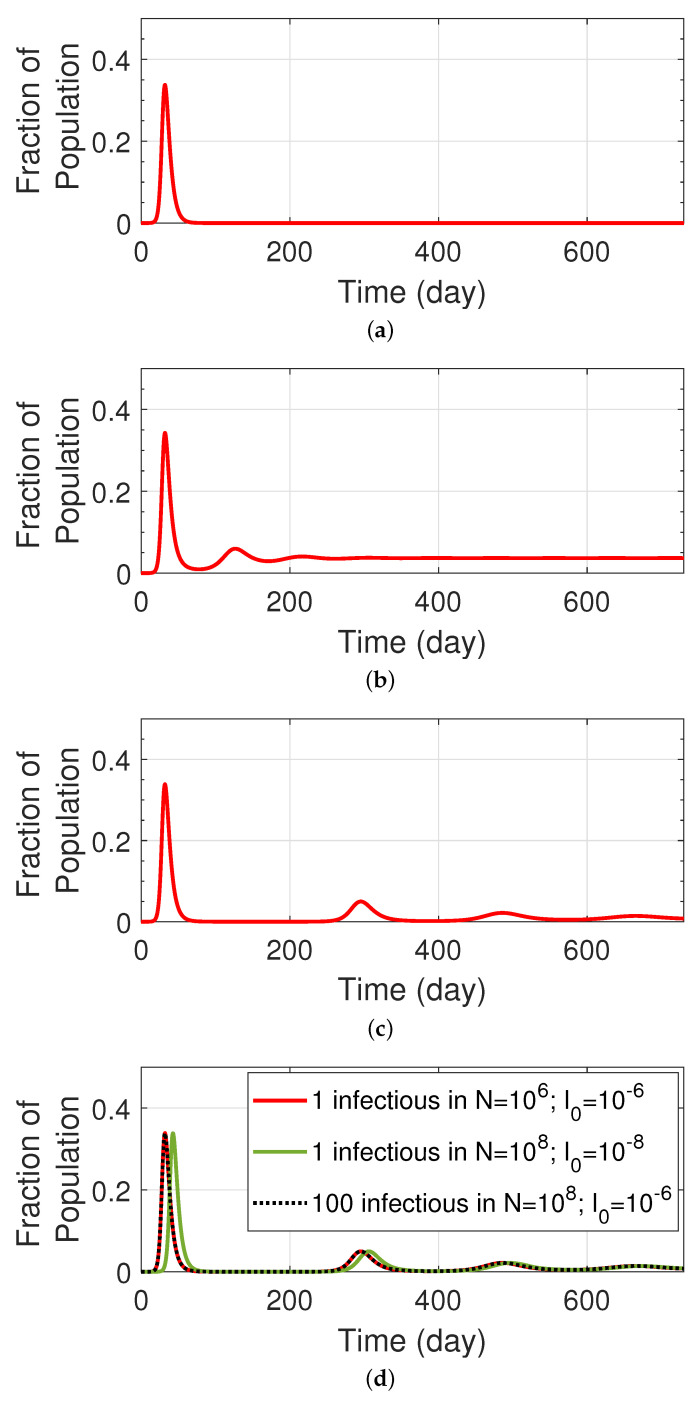
The fraction of infectious population for wild type virus projected by (**a**) classic SIR model; (**b**) classic SIRS model with 3 months natural immunity; (**c**) classic SIRS model with 12 months natural immunity; (**d**) classic SIRS model with different population sizes and different numbers of initial infectious individuals. Models are depicted in Figure 1.

Figure 2 displays the simulation results of the classic SIR and SIRS models for the projection of the fraction of infectious population, when only the wild type virus is considered. Table A1 shows the values of the rate parameters used in this simulation. As expected, the figure shows that the SIR attains permanent immunity for the entire community, which we know is not the case with the COVID-19. SIRS, on the other hand, predicts a temporary immunity depending on the duration of the natural immunity. When this period is short (Figure 2b), we can see that there is always a fraction of the population that remains infected. However, this fraction reduces significantly when there is an increased immunity period (Figure 2c), in which case subsequent waves of infected cases can be expected to follow the first wave. We then examined this scenario in a larger population of $100\times 10^{6}$ people with 1 and 100 infectious individuals, in Figure 2d. We can see that in comparison with our original scenario (1 infectious in a population of $10^{6}$), the larger population would experience a delay in all waves, if 1 infectious is assumed. However, in the case that the number of initially infectious individuals is increased to 100, we can observe the waves that are similar to our original scenario, basically because both scenarios result in an initial fractional infectious of $I_{0}=10^{-6}$. This basic observation is in line with what was witnessed both locally and globally during the early stages of the COVID-19 pandemic.

Over the course of the COVID-19 pandemic, the medical and public health communities have been able to identify many characteristics associated with the virus’s pandemic behavior that we were not aware of originally, and which warrant inclusion into epidemiological models. For example, since the start of the pandemic, we are now cognizant of the importance that asymptomatic infectious individuals have upon disease spread, the challenges associated with the development and deployment of multiple vaccines with various levels of efficacy and durability, as well as the emergence of new variants with higher transmissibility.

These and other observations necessitate introducing tailored extensions to the classic SIRS model in order to reflect and explore the more complex dynamics associated with each phenomenon. In the following sections, we present a variety of extensions to the classic SIRS model.

## 4. SIRS with Multiple Infection Severity Categories

**Figure 3:**
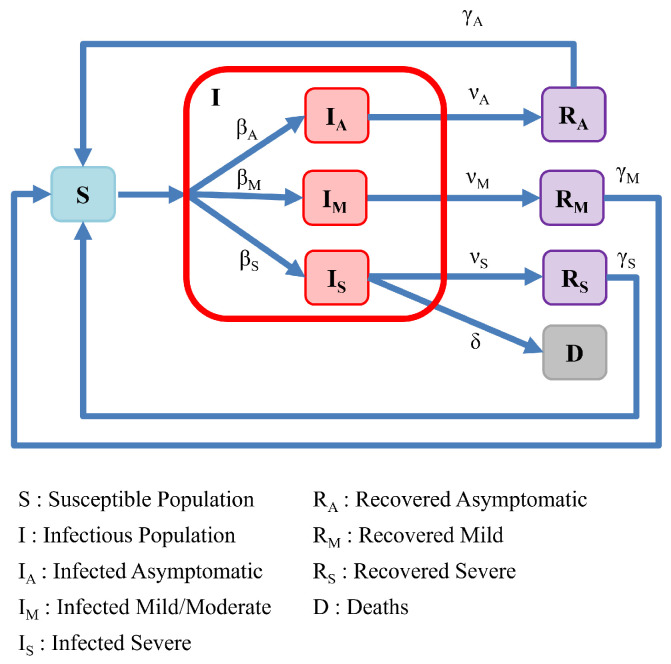
Model of population interactions with different levels of severity.

Our first extension to the classic SIRS model aims to introduce modifications that allow for a mathematical model that reflects different levels of disease severity. In Figure 3, we present a model with three different infectious population sub-groups, each representing a different severity level for the disease (asymptomatic, mild, and severe). We note that this model can be easily adapted to involve a different number of disease severity categories.

For the model depicted in Figure 3, when a susceptible individual (*S*) is in contact with any infectious individual (i.e., *I* corresponds to the total collection of infected individuals from all three categories), they may become infected with varying levels of severity. We assume that an individual contracts COVID to become an asymptomatic carrier of the disease with a rate of $\beta_{A}$, and thereby becomes a member of the $I_{A}$ subgroup of infected individuals. Likewise, an individual may contract COVID and experience a mild-to-moderate level of disease with a rate of $\beta_{M}$, and thereby becomes a member of the $I_{M}$ subgroup of infected individuals. Finally, an individual may contract a severe level of COVID with a rate of $\beta_{S}$, and thereby become a member of the $I_{S}$ subgroup of infected individuals. The parameter $\beta_{S}$ can also be interpreted as being proportionate to the fraction of the initially susceptible population that possesses underlying medical conditions (comorbidities) associated with a higher risk for serious COVID-19.

In the figure, we depict the entire population of infected individuals $I=I_{A}+I_{M}+I_{S}$, as well as the separate severity cases for infected individuals. Each subgroup of infected individuals is assigned different rate parameters governing their infection rates, recovery rates, and rates for returning to the susceptible population. However, as severe COVID-19 cases have often led to hospitalizations and death, the model also includes a class *D* corresponding to a deceased subpopulation.

For members of the asymptomatic subgroup $I_{A}$, we assume that they recover from their asymptomatic infection at a rate $\nu_{A}$. Likewise, the mild-to-moderate subpopulation recovers with a rate $\nu_{M}$, and the severely infected subgroup recovers with a rate $\nu_{S}$. Although not explicitly required in our model, it is often natural to consider $\nu_{A}>\nu_{M}>\nu_{S}$ since milder diseases should generally be associated with more rapid recovery. The rate at which severely infected patients suffer complications leading to death is associated with a rate parameter δ. Associated with the model just outlined, the following set of equations represents a dynamical system associated with an epidemiological model involving three levels of disease severity:

(3) $$\begin{array}{cc} \frac{dS}{dt} & =-\beta_{A}IS-\beta_{M}IS-\beta_{S}IS\\ \; & +\gamma_{A}R_{A}+\gamma_{M}R_{M}+\gamma_{S}R_{S},\\ \frac{dI_{A}}{dt} & =\beta_{A}IS-\nu_{A}I_{A},\\ \frac{dI_{M}}{dt} & =\beta_{M}IS-\nu_{M}I_{M},\\ \frac{dI_{S}}{dt} & =\beta_{S}IS-\nu_{S}I_{S}-\delta I_{S},\\ \frac{dR_{A}}{dt} & =\nu_{A}I_{A}-\gamma_{A}R_{A},\\ \frac{dR_{M}}{dt} & =\nu_{M}I_{M}-\gamma_{M}R_{M},\\ \frac{dR_{S}}{dt} & =\nu_{S}I_{S}-\gamma_{S}R_{S},\\ \frac{dD}{dt} & =\delta I_{S}, \end{array}$$

where $I=I_{A}+I_{M}+I_{S}$ is the entire population of infected individuals.

### Simulation Results and Discussions

An estimation of 29% [2] or almost one-third [21] of early strain cases were reported as asymptomatic, while in another study [22], it was reported that severe cases account for 5.2% of the total cases, which is consistent with the mean of hospitalized cases being in the range 0.4% to 9.2% as reported in [23]. However, it has also been noted in [2] that such public health statistics are highly uncertain given the numerous confounding factors affecting data collection and accuracy. For the purpose of this paper, though, we assume that 29% of COVID cases are asymptomatic, 5% are severe, and the remainder are symptomatic mild cases. The overall case fatality rate for symptomatic patients is estimated to be 1–2%, with an average period from onset of symptoms to death of 18 days [1,2,24].

**Figure 4:**
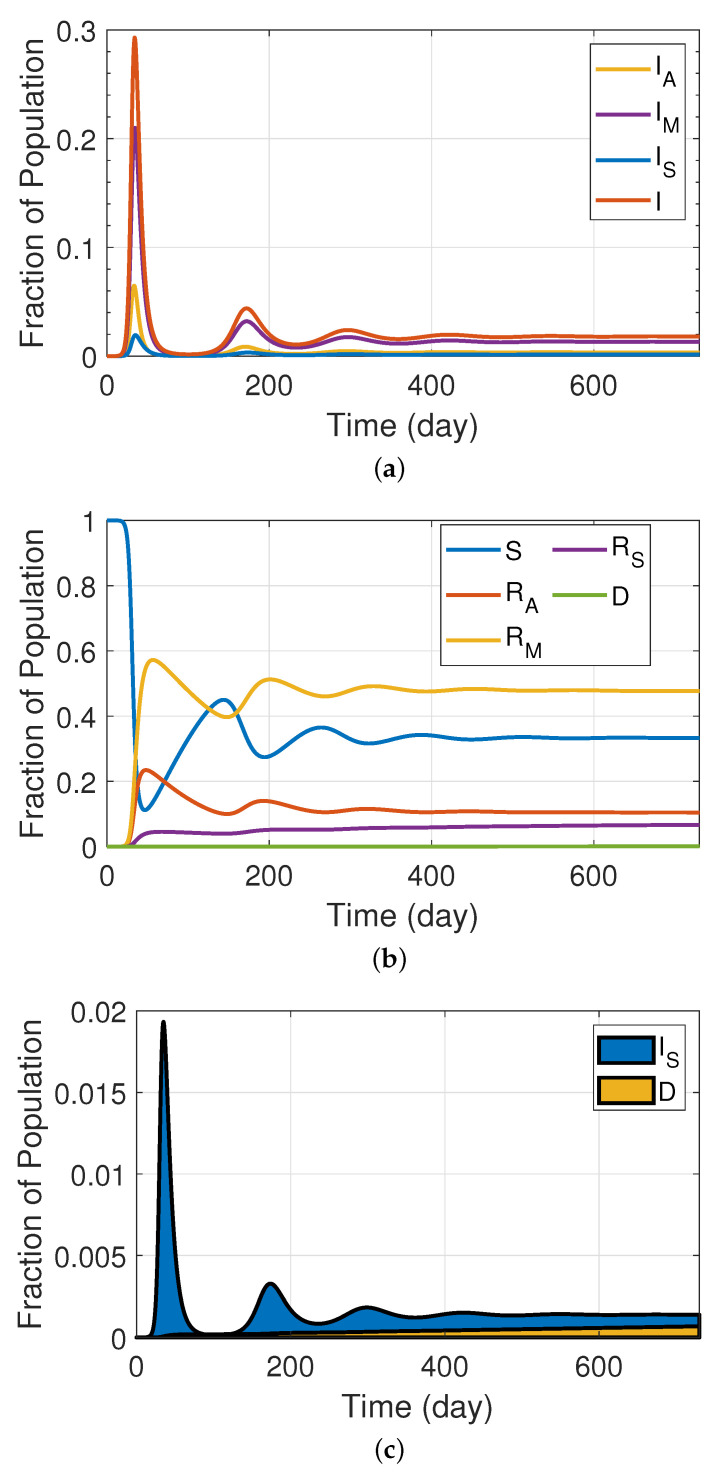
SIRS model with different levels of severity (model depicted in Figure 3). (**a**) Fraction of infectious population in asymptomatic, mild, and severe classes; (**b**) fraction of susceptible and recovered population in different groups; (**c**) fractional population of death compartment in comparison with severe population.

The simulation results for our extended SIRS model, which incorporates three levels of disease severity, is shown in Figure 4. Table A2 shows the values of the rate parameters we used in this simulation. Based on the discussion provided in the previous section, we used a period of 5 days for the period of infection for members of the mild subgroup, 3 days for asymptomatic patients, and 7 days for severe patients. Further, following the rationale that more severe disease corresponds to a proportionately deeper immune response involving more thorough training of the humoral immunity and, thereby, a more durable immune response, we considered a 3-month natural immunity period for asymptomatic individuals, 6 months for moderate patients, and 12 months for severe patients. The remaining parameters used in the simulations are set to the same values as the baseline values described in the preceding section. Figure 4a illustrates the proportion of the infected population associated with the asymptomatic, mild, and severe classes. Likewise, Figure 4b depicts the percentages associated with the susceptible and recovered population in various categories, while Figure 4c illustrates the fraction of the population resulting in death relative to the severe population. As can be seen, the mild population makes up the majority of infected individuals, resulting in the recovered mild group having the highest proportion.

## 5. SIRS with Multiple Severity Categories and Immunization

The availability of vaccines has been one of the most successful tools used for protecting public health and in preventing the spread of many diseases. Therefore, it is natural to explore how the availability of vaccines can be incorporated into epidemiological models.

**Figure 5:**
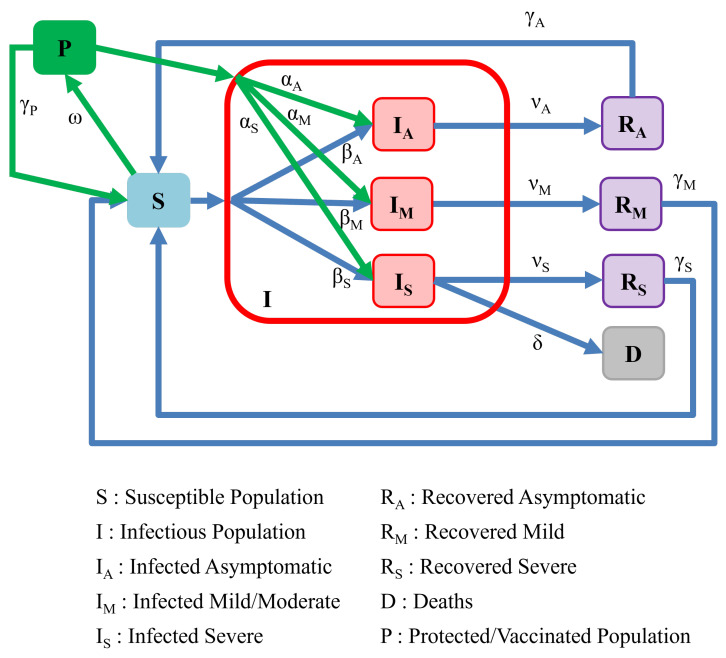
Compartmental model describing population interactions with different levels of disease severity and a vaccinated population.

The next extension to the SIRS model that we present is depicted in Figure 5. This model includes categories for different levels of disease severity and also incorporates a protected compartment associated with the subgroup of people who have received vaccination. The model assumes that susceptible individuals are vaccinated at rate ω(t) and then transferred to the protected compartment. We present a time dependent vaccination rate as formulated in the Equation (4), which includes the vaccination initiation time $t_{start}$. This equation assumes that the vaccination rate is 0 prior to time $t_{start}$, and that vaccination begins at time $t_{start}$ at the constant rate $\omega_{0}$. $\omega_{0}$ reflects the daily rate at which susceptible individuals are vaccinated, which in practice is affected by factors such as the vaccine supply, distribution, and administration constraints.

(4) $$\omega \left(t\right)=\left\{\begin{array}{cc} 0 & t<t_{start}\\ \omega_{0} & t\geq t_{start}. \end{array}\right.$$

Other forms of ω(t) can be easily introduced to suit the modeling needs.

Because vaccination confers a temporary protection, we include an outflow of people from the vaccinated subgroup to return to the susceptible compartment. This outflow is captured by the rate parameter $\gamma_{P}$, which is the inverse of vaccine durability. It is also worth noting that, because vaccination efficacy is not 100%, there is the potential that vaccinated persons will become infected at rates related to vaccine efficacy. We captured these cases using the rate parameters $\alpha_{A}$, $\alpha_{M}$, and $\alpha_{S}$.

The following set of differential equations represents the dynamical system associated with this model:

(5) $$\begin{array}{cc} \frac{dS}{dt} & =-\beta_{A}IS-\beta_{M}IS-\beta_{S}IS+\gamma_{A}R_{A}\\ \; & +\gamma_{M}R_{M}+\gamma_{S}R_{S}+\gamma_{P}P-\omega S,\\ \frac{dI_{A}}{dt} & =\beta_{A}IS+\alpha_{A}IP-\nu_{A}I_{A},\\ \frac{dI_{M}}{dt} & =\beta_{M}IS+\alpha_{M}IP-\nu_{M}I_{M},\\ \frac{dI_{S}}{dt} & =\beta_{S}IS+\alpha_{S}IP-\nu_{S}I_{S}-\delta I_{S},\\ \frac{dR_{A}}{dt} & =\nu_{A}I_{A}-\gamma_{A}R_{A},\\ \frac{dR_{M}}{dt} & =\nu_{M}I_{M}-\gamma_{M}R_{M},\\ \frac{dR_{S}}{dt} & =\nu_{S}I_{S}-\gamma_{S}R_{S},\\ \frac{dD}{dt} & =\delta I_{S},\\ \frac{dP}{dt} & =\omega S-\gamma_{P}P-\alpha_{A}IP-\alpha_{M}IP-\alpha_{S}IP, \end{array}$$

where $I=I_{A}+I_{M}+I_{S}$ represents the population of all infected individuals.

### Simulation Results and Discussion

Throughout most of the remainder of this paper, discussion involving vaccines and a vaccinated subpopulation will primarily draw upon early data associated with the Pfizer-BioNTech and Moderna mRNA vaccines, and the single-dose Janssen (Johnson & Johnson) vector virus vaccine against the wild type COVID-19 variant. When it is necessary for the discussion to examine questions related to disease variants and booster administration, we will utilize more recent vaccine data and will clearly reference the newer data and assumptions.

We will draw upon early-stage data regarding the efficacy of different vaccines in preventing disease corresponding to the manufacturers’ results involving a comparison between a placebo arm and vaccinated arm of a clinical trial. The Moderna vaccine has a 94.1% efficacy against the original COVID-19 strain of infection [25], while the Pfizer-BioNTech was reported to have a 95% efficacy [26], and the Janssen vaccine a 66.3% efficacy [27]. While [25,26,27] present the results of studies conducted by the manufacturers, similar results were corroborated through real world effectiveness studies. Notably, it is further reported in an independent comparative study [28] that the Moderna vaccine has a 93% effectiveness against COVID-19 hospitalizations, whereas the Pfizer-BioNTech vaccine has an 88% effectiveness and the Janssen vaccine has a 71% effectiveness against hospitalizations from the original COVID-19 variant. These levels of immunity are achieved 2 weeks after the second dose of the Moderna and Pfizer vaccines, or 2-weeks after a single dose of the Janssen vaccine. The Moderna vaccine employs a 28-day dose spacing, while the Pfizer vaccine schedule employs a 21-day dose spacing. After the first dose of Pfizer [26] reports a vaccine efficacy of 52.4%, beginning 14 days after dose 1 to an efficacy of 92.6% prior to dose 2. For the Moderna vaccine, [25] reports 93% efficacy during the period 14 days after dose 1 to dose 2. We assume a 6-month durability for Moderna and Pfizer vaccines and 2 months for Janssen vaccine. Additionally, in a key secondary study, [25] reports the efficacy of mRNA-1273 at preventing severe COVID-19. All the participants in the trial who had severe disease were in the placebo group indicating vaccine efficacy of 100%; however, because of the requirements for determining the 95% confidence interval, it could not be conclusively estimated as 1. Therefore in this section, we assume 99.99% efficacy for the sever group. We used the manufacturers efficacy data for parameter value selections in our studies, and report the values of the rate parameters we used in simulating this section in Table A3.

**Figure 6:**
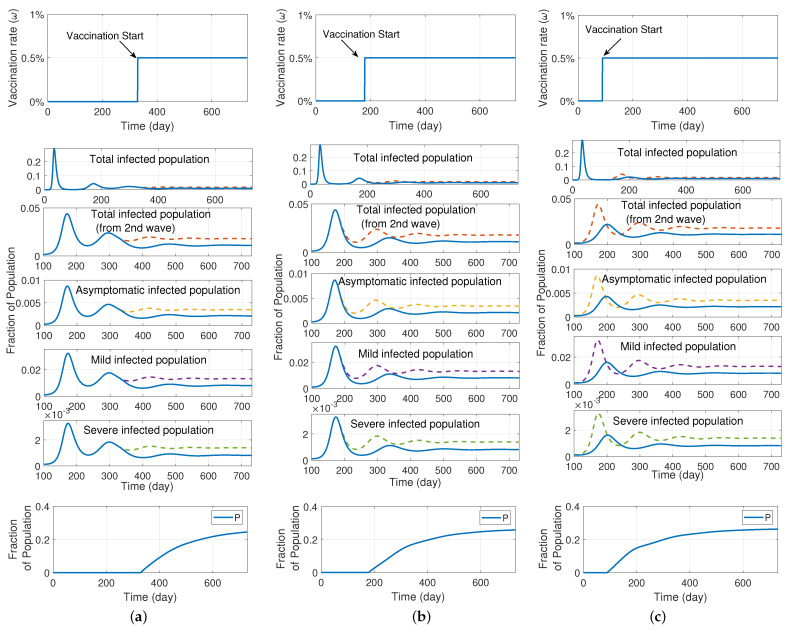
Results of the SIRS model with different levels of severity and vaccination (model depicted in Figure 5); investigating the effect of vaccination initiation on the second and subsequent waves of the pandemic assuming that 0.5% of the susceptible population is daily vaccinated; (**a**) vaccination starts after 11 months of pandemic; (**b**) vaccination begins after 6 months; (**c**) vaccination begins after 3 months. Dashed lines correspond to the model without vaccination, as shown in Figure 4a. The graphs in the last row show the fractional population of the protected individuals.

In Figure 6, we use the SIRS model with different levels of severity and vaccination to explore the impact of vaccination initiation on the second and subsequent waves of the pandemic. In this set of results, we assume that the vaccination rate ω(t) is 0 when $t<t_{start}$ and it equals 0.005 when $t\geq t_{start}$. This means that when vaccination initiates, 0.5% of the susceptible population is assumed to receive the vaccine daily. We examine the model for three different values for $t_{start}$ of 11 months, 6 months, and 3 months, and we use data from the Moderna vaccine for the vaccine efficacy. Figure 6a shows the results when vaccination begins 11 months after the start of the pandemic, while Figure 6b,c assume that vaccination begins 6 months and 3 months after the onset of the pandemic, respectively. Dashed lines correspond to the model without vaccination, which are the same plots as in Figure 4a. The first row of the figure shows the vaccination rate for different vaccination start times. The second row of graphs depict the infected population throughout the pandemic with the x-axis representing a time range from day 0 to day 730, during 2 years of the pandemic. It is important to recognize that the first peak of the graph corresponds to the first wave of the infection, which in the simulation occurs prior to the deployment of vaccination. The graphs in the last row depict the fraction of protected population in each vaccination scenario.

**Figure 7:**
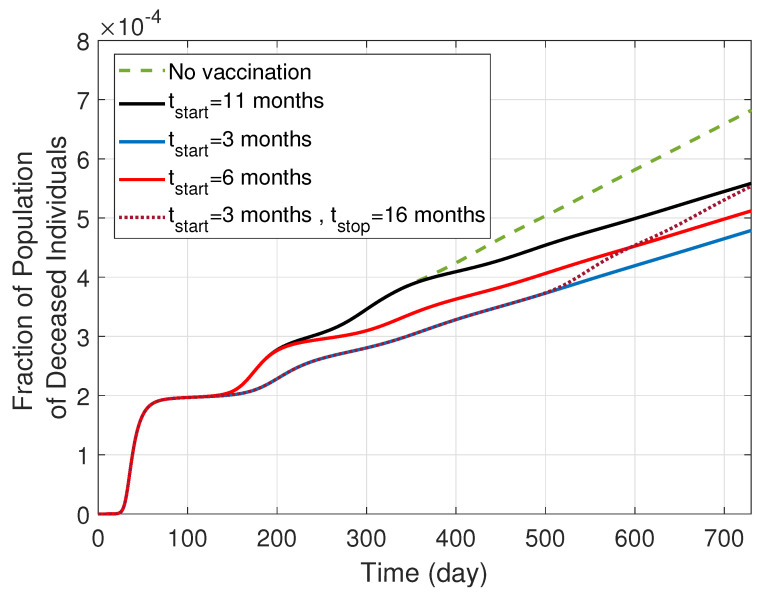
The total deceased in the SIRS model with different levels of severity and vaccination (i.e., the model depicted in Figure 5). Earlier vaccination leads to reduced deaths, and sustained vaccination ensures that the mortality rate is less than halting vaccination.

We were interested in exploring the potential impact that earlier or later vaccination could have had on the population dynamics. In order to facilitate this, the third, fourth, fifth, and sixth rows of the figure are zoomed in to focus on the second and subsequent waves of the pandemic. A comparison of panels a, b, and c shows that early immunization would result in weaker pandemic waves in all levels of the infection. The effect of vaccination initiation on the number of deaths is then compared in Figure 7. This figure illustrates the effects that early vaccination would have had on reducing the number of deaths from COVID-19. Earlier vaccination leads to deceased curves that are shifted lower, corresponding to fewer deaths. However, it is also noteworthy to observe the slope of the unvaccinated trend versus all of the vaccinated trend lines. The slope for all of the vaccinated cases is less steep than the unvaccinated trend, and illustrates the importance of the entire population continuing to vaccinate. Continued vaccination provides a persistent protected category of individuals, and should the population refrain from continuing to vaccinate, the death trend would return to having the same slope as the unvaccinated trend, as can be seen by the dotted line in Figure 7. However, we note that in real-world COVID-19 situations, death rates are driven by a range of factors in addition to vaccination status, including age, pre-existing medical conditions, and gender.

## 6. SIRS with Multiple Vaccines

**Figure 8:**
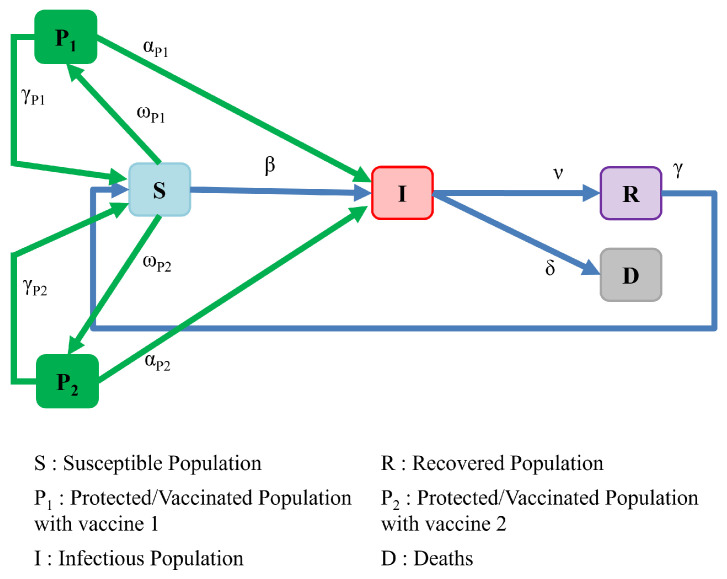
Model of population interactions with different vaccine types.

In our next SIRS-based extension, we model the effect of deploying a vaccination strategy that employs multiple vaccines of varying immunization efficacy and durability, as depicted in Figure 8. We represent the entire infectious population as a single compartment *I* and suppose that susceptible individuals are immunized at variable rates with either of the two accessible vaccines $P_{1}$ and $P_{2}$. The two vaccination rates $\omega_{P1}\left(t\right)$ and $\omega_{P2}\left(t\right)$ can depend on many factors, including pragmatic issues related to supply production, vaccine distribution and storage, as well as the quantity of doses required to achieve a vaccine’s target efficacy.

The dynamics of this model is represented by the set of equations below:

(6) $$\begin{array}{cc} \frac{dS}{dt} & =-\beta IS+\gamma R+\gamma_{P1}P_{1}\\ \; & +\gamma_{P2}P_{2}-\omega_{P1}S-\omega_{P2}S,\\ \frac{dI}{dt} & =\beta IS+\alpha_{P1}IP_{1}+\alpha_{P2}IP_{2}-\nu I-\delta I,\\ \frac{dR}{dt} & =\nu I-\gamma R,\\ \frac{dD}{dt} & =\delta I,\\ \frac{dP_{1}}{dt} & =\omega_{P1}S-\gamma_{P1}P_{1}-\alpha_{P1}IP_{1},\\ \frac{dP_{2}}{dt} & =\omega_{P2}S-\gamma_{P2}P_{2}-\alpha_{P2}IP_{2}, \end{array}$$

Extensions to this model that involve more than two vaccines are straightforward.

### Simulation Results and Discussion

**Figure 9:**
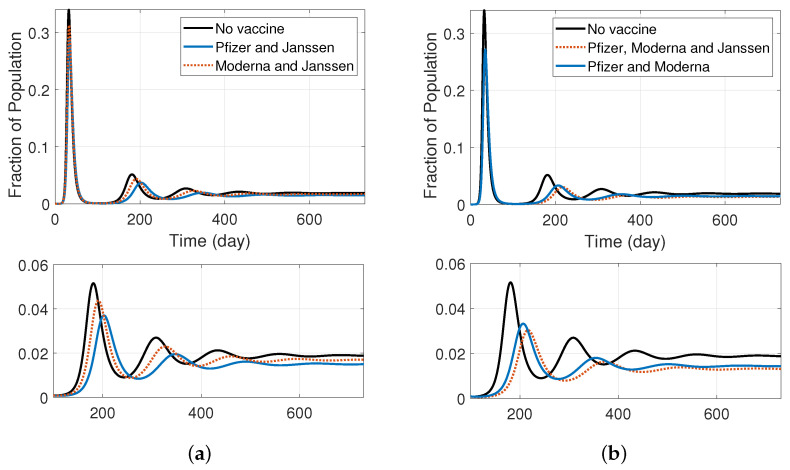
SIRS model with different vaccine types (model depicted in Figure 8); comparing the effect of vaccinating with various vaccines assuming that the average daily rate of vaccination by Pfizer is 0.3%, by Moderna is 0.15%, and by Janssen is 0.03%; (**a**) fraction of infectious population when individuals are vaccinated by Moderna and Janssen or Pfizer and Janssen; (**b**) fraction of infectious population when people are vaccinated by Moderna and Pfizer or by all three vaccines. Magnified versions of figures in the top row are represented in the second row (from the second wave).

We were interested in understanding the impact that employing more than a single vaccine type could have on the disease trajectory. Figure 9 shows the results of the SIRS model with two vaccine types (i.e., the model shown in Figure 8). Table A4 shows the values of the rate parameters used in this simulation.

We used infection parameters and vaccine-related parameters specified in Section 3 and Section 5, respectively. Based on the daily doses given by the manufacturer reported in [29], we assume that an average of 0.3% are being vaccinated daily by Pfizer, and average of 0.15% are vaccinated daily by Moderna, and on average 0.03% of the susceptible population are daily vaccinated by the Janssen vaccine. The beginning of the vaccination is assumed as the time reference (day 0) in all graphs. Figure 9a shows the infectious population fraction when the population is vaccinated with Pfizer and Janssen, or Moderna and Janssen vaccines, and (b) shows the fractional infectious population when susceptible individuals are vaccinated with Pfizer and Moderna or all three available vaccines. The second row depicts magnified versions of the first row graphs, focusing on the second wave and its subsequent waves. As can be seen, the vaccination rate (as a result of vaccine availability and distribution) is a major contributor for the timing and peak of the second wave and its subsequent waves. When comparing figures (a) and (b), it can be seen that using two vaccines that are more widely available and effective (Pfizer and Moderna versus Pfizer and Janssen) results in fewer infections in the long run. Vaccinations with all three available vaccines also result in lower peaks over the course of the pandemic. In the next section, we will expand the model to include multiple doses of a vaccine.

## 7. SIRS with Multi-Dose Immunization and Vaccinated and Unvaccinated Compartments

Over the course of the pandemic, one of the major questions that the public health community has been facing is centered on how and when multiple doses should be deployed for the Pfizer and Moderna mRNA vaccines. In particular, initial deployments involved a two-dosage strategy, which eventually was updated to three doses for select subgroups of the population (e.g., elderly or immunocompromised). Questions, such as when to initiate the third dose, and the implications should a third dose reduce the availability for first and second shots, have been at the forefront of the public health discussion.

**Figure 10:**
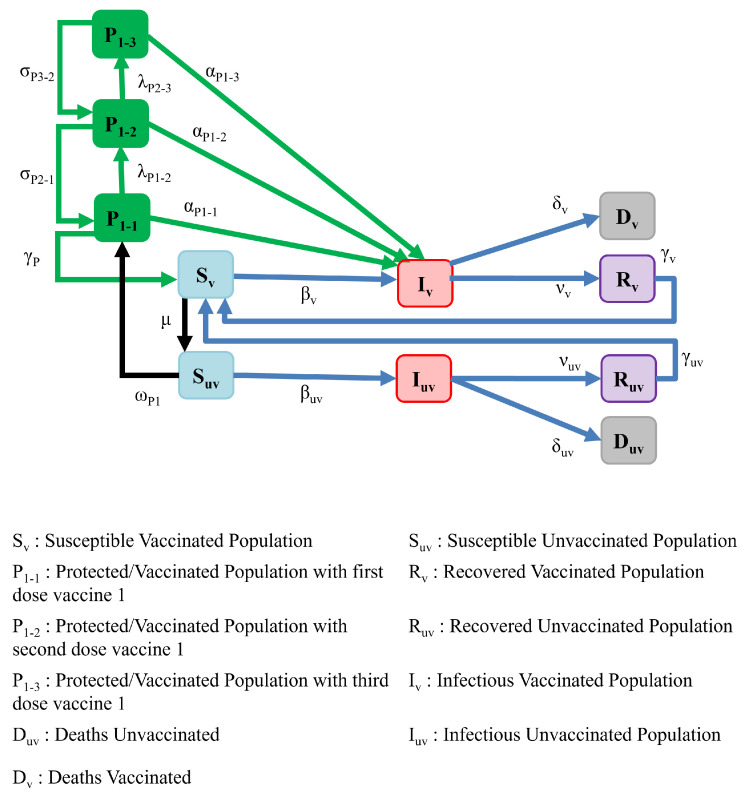
Model of multi-dose immunization and vaccinated and unvaccinated compartments.

To examine such questions, in this section, we extend the SIRS model to capture immunization with multiple doses, while including separate compartments for vaccinated and unvaccinated individuals. Figure 10 depicts our model, which reflects the various susceptible, infectious, and recovered compartments given their immunization situation. It is assumed that a three-shot vaccine will be used. $P_{1-1}$, $P_{1-2}$, and $P_{1-3}$ contain the populations of protected individuals after the first, second, and third doses, respectively. $\lambda_{P1-2}$ and $\lambda_{P2-3}$ represent the rate at which the vaccinated people receive their second and third shots, respectively. These time dependent vaccination rates are formulated as in Equation (7) to include the second and third dose vaccination times $t_{V2}$ and $t_{V3}$. Constant rates are assumed after the initiation of the second and third doses of vaccination.

(7) $$\begin{array}{ccc} \lambda_{P1-2}\left(t\right)=\left\{\begin{array}{cc} 0 & t<t_{V2}\\ \lambda_{0} & t_{V2}\leq t\leq t_{V3}\\ \lambda_{1} & t\geq t_{V3} \end{array}\right. & , & \lambda_{P2-3}\left(t\right)=\left\{\begin{array}{cc} 0 & t<t_{V3}\\ \lambda_{2} & t\geq t_{V3} \end{array}\right. \end{array}$$

This model reflects the efficacy of each dose of the vaccine (represented by $\alpha_{P1-1}$, $\alpha_{P1-2}$, and $\alpha_{P1-3}$) and immunity period after the first, second, and third doses (represented by $\gamma_{P}$, $\sigma_{P2-1}$, and $\sigma_{P3-2}$). As can be seen in our model, we assume that the waning of protection for a person receiving three doses of a vaccine is modeled as a step down from compartment $P_{1-3}$ to compartment $P_{1-2}$, which has less protection from infection than the prior compartment $P_{1-3}$, as reflected by the assumption that $\alpha_{P1-2}>\alpha_{P1-3}$). Similarly, the waning of protection for two doses of a vaccine is modeled as a step down from compartment $P_{1-2}$ to $P_{1-1}$.

Further, it is assumed that unvaccinated susceptible individuals will be vaccinated at a rate $\omega_{P1}$ and will be moved to the protected compartment ($P_{1-1}$). We assume different constant levels for $\omega_{P1}$ as presented in the following equation in order to reflect the vaccine supply being divided amongst first, second, and third doses.

(8) $$\omega_{P1}\left(t\right)=\left\{\begin{array}{cc} \omega_{0} & t<t_{V2}\\ \omega_{1} & t_{V2}\leq t\leq t_{V3}\\ \omega_{2} & t\geq t_{V3} \end{array}\right.$$

Vaccinated individuals will lose the initial potency of their vaccinations and transit to a separate susceptible category, which we refer to as the vaccinated susceptible compartment ($S_{v}$). The purpose of introducing this extra compartment is to reflect a minimal level of protection due to the durability of the first dose. It is also assumed that susceptible vaccinated individuals would move to the unvaccinated (and, hence, susceptible) compartment, after a period of 1/μ. Additionally, it is assumed that any unvaccinated individuals who have already been infected, migrate to the vaccinated susceptible compartment, after recovery. In other words, prior infection is regarded as conferring some baseline level of immunity.

The dynamics of this model is represented by the set of differential equations below:

(9) $$\begin{array}{cc} \frac{dS_{v}}{dt} & =-\beta_{v}\left(I_{v}+I_{uv}\right)S_{v}+\gamma_{v}R_{v}+\gamma_{P}P_{1-1}\\ \; & +\gamma_{uv}R_{uv}-\mu S_{v},\\ \frac{dI_{v}}{dt} & =\beta_{v}\left(I_{v}+I_{uv}\right)S_{v}+\alpha_{P1-1}I_{v}P_{1-1}\\ \; & +\alpha_{P1-2}I_{v}P_{1-2}+\alpha_{P1-3}I_{v}P_{1-3}-\nu_{v}I_{v}-\delta_{v}I_{v},\\ \frac{dR_{v}}{dt} & =\nu_{v}I_{v}-\gamma_{v}R_{v},\\ \frac{dD_{v}}{dt} & =\delta_{v}I_{v},\\ \frac{dP_{1-1}}{dt} & =\omega_{P1}S_{uv}-\gamma_{P}P_{1-1}-\alpha_{P1-1}I_{v}P_{1-1}\\ \; & -\lambda_{P1-2}P_{1-1}+\sigma_{P2-1}P_{1-2},\\ \frac{dP_{1-2}}{dt} & =-\sigma_{P2-1}P_{1-2}-\alpha_{P1-2}I_{v}P_{1-2}\\ \; & +\lambda_{P1-2}P_{1-1}-\lambda_{P2-3}P_{1-2}+\sigma_{P3-2}P_{1-3},\\ \frac{dP_{1-3}}{dt} & =-\sigma_{P3-2}P_{1-3}-\alpha_{P1-3}I_{v}P_{1-3}\\ \; & +\lambda_{P2-3}P_{1-2},\\ \frac{dS_{uv}}{dt} & =-\beta_{uv}\left(I_{v}+I_{uv}\right)S_{uv}-\omega_{P1}S_{uv}+\mu S_{v}\\ \frac{dI_{uv}}{dt} & =\beta_{uv}\left(I_{v}+I_{uv}\right)S_{uv}-\nu_{uv}I_{uv}-\delta_{uv}I_{uv},\\ \frac{dR_{uv}}{dt} & =\nu_{uv}I_{uv}-\gamma_{uv}R_{uv},\\ \frac{dD_{uv}}{dt} & =\delta_{uv}I_{uv}, \end{array}$$

### Simulation Results and Discussion

**Figure 11:**
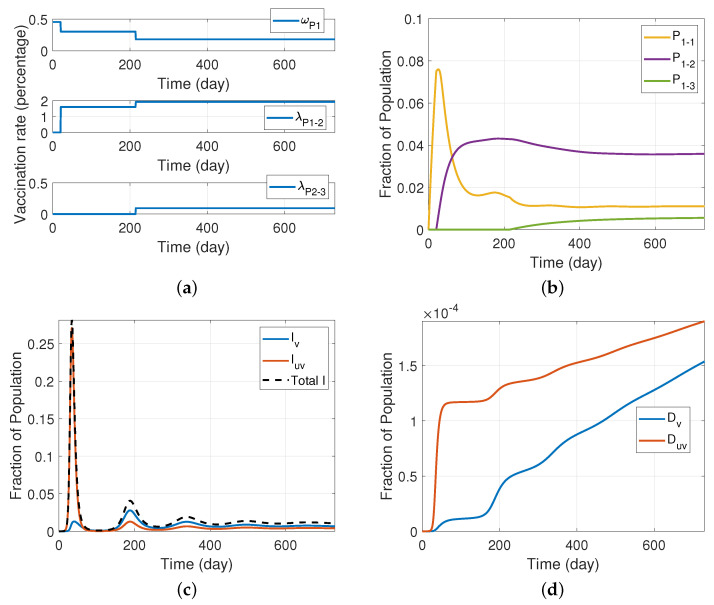
An SIRS model with a three-dose vaccine and different vaccinated and unvaccinated compartments (model depicted in Figure 10), assuming that vaccination with the Pfizer vaccine is available from the beginning; (**a**)vaccination rates of first dose, second dose, and third dose from top row to bottom row, respectively; (**b**) fraction of individual protected by one, two, and three doses of a vaccine; (**c**) fraction of infectious individual; (**d**) fraction of deceased individuals.

Figure 11 depicts our simulation results for the SIRS model with a three-dose vaccine and various vaccinated and unvaccinated compartments described in Figure 10. We assume that the vaccination is available from the beginning in this set of results, and we use Pfizer vaccine efficacy specifications. Table A5 shows the values of the rate parameters we used in this section. In addition to the assumptions given in Section 5, we assume that a fixed amount of vaccine supply is divided across those receiving their first, second, and third doses. As shown in Figure 11a, this results in different time-dependent vaccination rates. Given these vaccination rates, Figure 11b shows the fraction of the population vaccinated by first, second, and third doses, whereas panel (c) shows the fraction of infectious population. Because every infected individual moves to the susceptible vaccinated compartment after recovery, the population of $S_{V}$ grows, eventually leading to an increase in the second wave and subsequent waves of $I_{V}$. Figure 11d, on the other hand, shows that the fractional deceased population of vaccinated people is lower than that of unvaccinated people, reiterating the benefit of vaccines in saving lives. We also note that, in these simulations, we assumed that vaccinated individuals move to the susceptible vaccinated compartment after two months, and those who are vaccinated by the second dose move to $P_{1-1}$ compartment after six months. We also supposed that the population of compartment $P_{1-3}$ shifts down to compartment $P_{1-2}$ after 6 months following the booster (third) shot, and that after 6 months, the population of compartment $S_{v}$ transfers to the unvaccinated and susceptible compartment $S_{uv}$.

**Figure 12:**
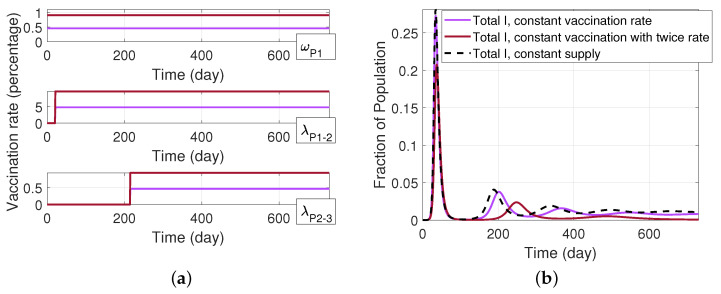
SIRS model with a three-dose vaccine and different vaccinated and unvaccinated compartments (model depicted in Figure 10), assuming that the vaccination with the Pfizer vaccine is available from the beginning with sufficient supply for vaccinating all groups; (**a**) constant vaccination rates of the first dose, second dose, and third dose from the top row to bottom row, respectively, each for two different values; (**b**) fraction of infectious individual.

**Figure 13:**
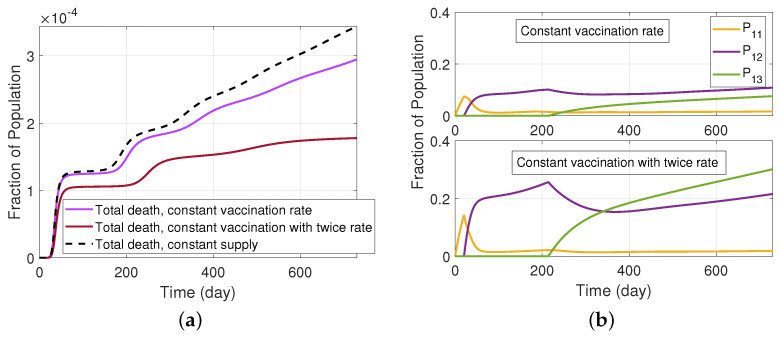
(**a**) The total deceased fractional population in the SIRS model with a three-dose vaccine (model depicted in Figure 10). We compare the assumption that there is sufficient vaccination supply with the Pfizer vaccine to maintain a constant rate of vaccination for those receiving first, second, and third doses in comparison to maintaining a constant total vaccine supply across all groups. (**b**) Fraction of individuals protected by one, two, and three doses of a vaccine with constant vaccination rates.

Next, we examine the potential benefit that can arise when we increase the vaccine supply. Specifically, contrary to the assumption in Figure 11a in which there is a constrained vaccine supply that is proportionately divided among those receiving first, second, and third doses, we instead assume that it is possible for vaccine production and distribution to maintain a fixed vaccination rate for first, second, and third doses, even as a larger fraction of the population achieves a boosted state. To examine this scenario, we assume a constant rate $\omega_{P1}$ for those receiving their first dose of the Pfizer vaccine. Then, at time $t_{V2}$, we assume a fixed rate $\lambda_{P1-2}$ for those receiving their second dose of the Pfizer vaccine. Finally, at time $t_{V3}$, we assume a fixed rate $\lambda_{P2-3}$ for individuals in $P_{1-2}$ to receive their third dose of the Pfizer vaccine. In Figure 12, we consider a case with a baseline set of rates $\left(\omega_{P1},\lambda_{P1-2},\lambda_{P2-3}\right)$ and twice those rates, i.e., $\left(2\omega_{P1},2\lambda_{P1-2},2\lambda_{P2-3}\right)$. For these cases, Figure 12a depicts the associated vaccination rates, while Figure 12b depicts the resulting infectious population. As can be seen from the graphs, when compared to the initial scenario of Figure 11, increasing and maintaining the vaccination rates leads to a further reduction in the infectious population. In order to better explore the implication of increasing the vaccine supply as the population progresses from first, to second, and third vaccine doses, we present the total deceased population fraction in Figure 13a. As can be seen in this graph, there is a dramatic benefit that results from increasing the vaccine supply to progressively move more of the population into the boosted (third dose) compartment, while continuing efforts to administer first doses of the vaccine. In particular, we can see in the case where we doubled the vaccine rates that the deceased population fraction has plateaued near the end of the two year time period. Figure 13b shows the fraction of the population that are vaccinated by one, two, and three doses in this scenario. It is seen in the graph that by increasing the vaccination rates the number of individuals that are receiving their third dose is increasing, and thereby leading to significantly reduced death rates.

Finally, we should note that the use of the parameters $\left(\omega_{P1},\lambda_{P1-2},\lambda_{P2-3}\right)$ can allow one to explore a wide array of factors associated with the vaccination effort and its impact upon how a disease progresses through the population. For example, it is possible to explore the effects of vaccine saturation and hesitancy in our model by multiplying the time-varying parameters $\left(\omega_{P1},\lambda_{P1-2},\lambda_{P2-3}\right)$ with a suitable time-varying weighting function that reflects the dampening in vaccine uptake. As an example, one can utilize a sigmoidal function of the form

$$f\left(t\right)=1-\frac{1}{1+e^{-c(t-\tau )}}$$

to capture a decrease in the overall vaccination effort as time progresses (time t=τ being the cross-over point where f(τ)=0.5) and where eventually the entire population will stop vaccinating.

## 8. SIRS with Multiple Variants and Immunization

**Figure 14:**
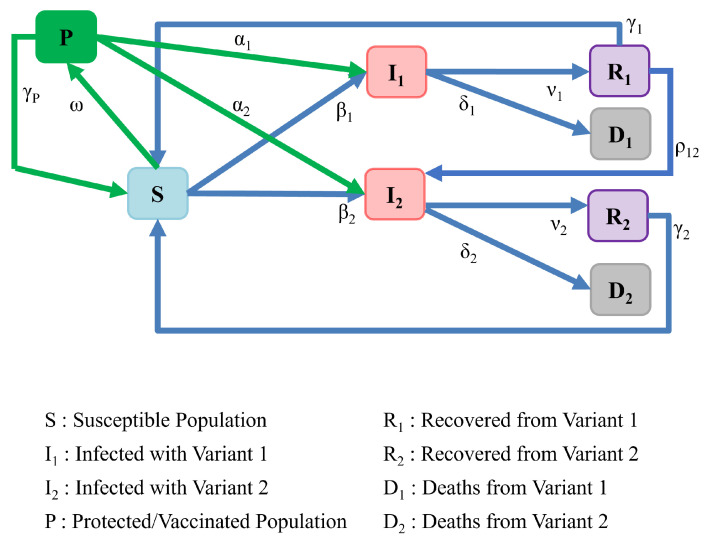
Model of population interactions with different variants, and vaccinated population.

Viruses such as SARS-CoV-2 are constantly evolving, and we have seen numerous variants since the pandemic began. At the time of writing, the Centers for Disease Control and Prevention (CDC) is monitoring 12 variants, which include the most recent two, Delta and Omicron, which are variants of concern [30]. Therefore, we extend the SIRS model in this section to account for the circulation of these two recent variants as well as the impact of vaccination on them. Figure 14 depicts our SIRS model extension, which accounts for these two variants and a single vaccine with varying effectiveness against these two different variants. The model implies that, following recovery from variant 1 (e.g., the Delta variant), there is a chance of contracting variant 2 (e.g., the Omicron variant). As a result, a path from compartment $R_{1}$ to compartment $I_{2}$ has been introduced in Figure 14. However, we note that we have not introduced a path from compartment $R_{2}$ to $I_{1}$ because recent results in the scientific literature suggest that infection with the omicron variant provides powerful and extended resistance to infection by the delta variant [31].

The dynamics of this model is represented by the set of equations below:

(10) $$\begin{array}{cc} \frac{dS}{dt} & =-\beta_{1}I_{1}S-\beta_{2}I_{2}S+\gamma_{1}R_{1}\\ \; & +\gamma_{2}R_{2}+\gamma_{P}P-\omega S,\\ \frac{dI_{1}}{dt} & =\beta_{1}I_{1}S+\alpha_{1}I_{1}P-\nu_{1}I_{1}-\delta_{1}I_{1},\\ \frac{dR_{1}}{dt} & =\nu_{1}I_{1}-\gamma_{1}R_{1}-\rho_{1-2}R_{1}I_{2},\\ \frac{dI_{2}}{dt} & =\beta_{2}I_{2}S+\alpha_{2}I_{2}P-\nu_{2}I_{2}-\delta_{2}I_{2}+\rho_{1-2}R_{1}I_{2},\\ \frac{dR_{2}}{dt} & =\nu_{2}I_{2}-\gamma_{2}R_{2},\\ \frac{dD_{1}}{dt} & =\delta_{1}I_{1},\\ \frac{dD_{2}}{dt} & =\delta_{2}I_{2},\\ \frac{dP}{dt} & =\omega S-\gamma_{P}P-\alpha_{1}I_{1}P-\alpha_{2}I_{2}P. \end{array}$$

### Simulation Results and Discussion

In addition to the assumptions for the wild type variant provided in Section 3 and vaccination efficacy provided in Section 5, we employed different parameters for the Delta and Omicron variants of concern. While the Delta variant has an $R_{0}$ of just under 7, the $R_{0}$ of the Omicron variant was estimated to be as high as 10 [32]. Moreover, according to [33], we assume that two doses of the Moderna vaccine is 86.7% effective against infection with the Delta variant, while there is reduced effectiveness against Omicron and, therefore, we used a 50% vaccine efficacy against Omicron in our simulations. Table A6 shows the values of the rate parameters we used in the simulations.

**Figure 15:**
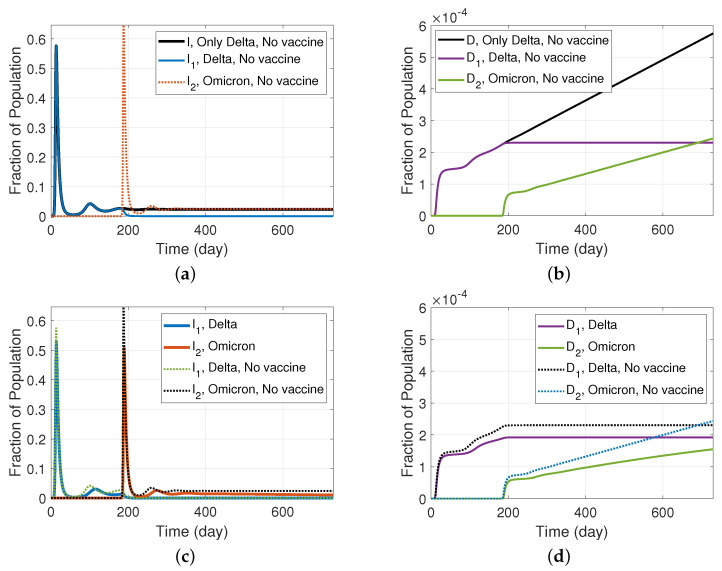
Results for an SIRS model with two variants and a vaccine with different efficacy (model depicted in Figure 14); (**a**) fraction of infectious population and (**b**) fraction of deceased population, when Omicron appears half-way through Delta, in comparison with a case with only delta. (**c**) Fraction of infectious population and (**d**) fraction of deceased population, when Omicron appears half-way through Delta, assuming that a vaccine with different effectiveness on Delta and Omicron is available from the beginning, and the vaccination rate is 0.5%.

The simulation results for our modified SIRS model with two variants, and employing a vaccine with different effectiveness against each variant, are shown in Figure 15. It was assumed that the vaccine is accessible from the beginning and that 0.5% of the susceptible population are vaccinated on a daily basis. We initiated the introduction of Omicron 6 months after Delta, and examined the infectious population fraction and the fraction of the total population that was deceased, which are reflected in panels (a) and (b), respectively. The graphs are compared to a hypothetical situation in which Delta is the only variant in circulation. Reflecting the higher $R_{0}$ value for Omicron, we see that when Omicron appears, it becomes the dominant variant after the initial wave. This is consistent with real-world statistics, and the global trend that has been observed at the start of 2022. However, because Omicron infections are assumed 91% less lethal than Delta infections [34], we can see in panel (b) that the Omicron’s rapid rise and the associated rapid decline in Delta’s prevalence leads to almost no new deaths to delta-caused deaths. Likewise, the fact that the death rate caused by Omicron is much lower, leads to a less steep death curve in spite of Omicron being the surviving, dominant variant. The effects of vaccination on the infectious population and death fractions are investigated in panels (c) and (d), respectively. The graphs reconfirm that vaccination reduces infectious populations and, more importantly, the rate of disease-related deaths.

## 9. Conclusions

In this paper, we explored a variety of aspects associated with the COVID-19 pandemic through the lens of epidemiological modeling. This survey includes models that study the severity of the disease, those that explore the emergence of new variants, those that investigate the impacts of vaccination strategies, and those that look into the impact of different social behaviors on pandemic trends, such as social distancing and lockdowns. Building upon the methods presented in our survey, we proposed a collection of useful compartmental models that extend the classical SIRS model, allowing to further analyze the dynamics that arise in the presence of different levels of disease severity, capturing the effects of vaccinations on different subgroups of infected populations, as well as different vaccines with different levels of effectiveness, modeling the impact of a vaccine strategy involving multiple doses, yielding progressively-enhanced effectiveness and durability, allowing one to investigate pandemic trends as new variants emerge, in combination with a corresponding decrease in vaccine effectiveness to emergent variants. We used publicly available real-world data and applied parameters associated with the wild type variant for many of our models, as early pandemic data are now widely available. Further, for our studies related to multiple co-existing variants, we used specifications for the newer Delta and Omicron variants of concern. In our simulations, we also assumed vaccine data from Moderna, Pfizer, and Janssen. When the natural immunity interval is short, our simulated results show that a steady-state condition will arise in which a fraction of the population is consistently infected. Our findings also show that mild infectious groups make up the majority of infected people, and that early immunization causes pandemic waves to be weaker across all illness severity levels, and the number of disease-caused deaths to be lower. Our results also show that a three-dose vaccine has a strong chance of reducing the infectious population when dealing with a single variant and having access to a highly efficient vaccine. When a new variant with lower vaccination efficacy emerges, however, it quickly becomes the dominant circulating variant, which is consistent with observations associated with the Omicron variant out-competing the Delta variant. Finally, we should note that the models that we have presented are quite flexible and can allow the public health modeler to explore many problems that were not directly examined in this paper. Notably, evolving trends in vaccination efforts, such as the impact of vaccine saturation and hesitancy on how the disease propagates through a population, can be explored using simple modifications to our compartmental models.

## Figures and Tables

**Table 1:** Descriptions of the basic transition rates for all models.

Notation	Description
β	Transition rate from susceptible compartment to infectious compartment.
ν	Transition rate from infectious compartment to recovered compartment.
γ	Transition rate from recovered compartment to susceptible compartment.
δ	Transition rate from infectious compartment to death compartment.
ω	Transition rate from susceptible compartment to vaccinated compartment.
$\gamma_{P}$	Transition rate from vaccinated compartment to susceptible compartment.
α	Transition rate from vaccinated compartment to infectious compartment.
$\lambda_{P}$	Transition rate from a previously vaccinated to a newly vaccinated compartment.
$\sigma_{P}$	Transition rate from a newly vaccinated to a previously vaccinated compartment.
μ	Transition rate from vaccinated susceptible to unvaccinated. susceptible compartment.
ρ	Transition rate from recovered compartment to infectious compartment.

## Data Availability

The data presented in this study are available in the article.

## Abbreviations

The following abbreviations are used in this manuscript:

COVID-19	coronavirus disease 2019
SARS-CoV-2	severe acute respiratory syndrome coronavirus 2
SIR	susceptible-infectious-recovered
SIRS	susceptible-infectious-recovered-susceptible
SEIR	susceptible-exposed-infectious-recovered

## Appendix A. Parameter Values

This appendix contains the values of the rate parameters used in the simulation results presented in this paper.

**Table A1:** Rate parameters used for Figure 2.

β	γ	ν
0.664	0.0111, 0.0028	0.2

**Table A2:** Rate parameters used for Figure 4.

$\beta_{A}$	$\beta_{M}$	$\beta_{S}$	$\gamma_{A}$	$\gamma_{M}$	$\gamma_{S}$	$\nu_{A}$	$\nu_{M}$	$\nu_{S}$	δ
0.1926	0.4382	0.0332	0.0111	0.0056	0.0028	0.3333	0.2	0.1429	$5.556\times 10^{-4}$

**Table A3:** Rate parameters used for Figure 6 and Figure 7.

$\beta_{A}$	$\beta_{M}$	$\beta_{S}$	$\gamma_{A}$	$\gamma_{M}$	$\gamma_{S}$	$\nu_{A}$	$\nu_{M}$	$\nu_{S}$	δ	$\gamma_{P}$	$\alpha_{A}$	$\alpha_{M}$	$\alpha_{S}$	$\omega_{0}$
0.1926	0.4382	0.0332	0.0111	0.0056	0.0028	0.3333	0.2	0.1429	$5.556\times 10^{-4}$	0.0056	0.0114	0.0259	$3.32\times 10^{-6}$	0.005

**Table A4:** Rate parameters used for Figure 9.

β	γ	$\gamma_{P1}$	$\gamma_{P2}$	$\omega_{P1}$	$\omega_{P2}$	ν	$\alpha_{P1}$	$\alpha_{P2}$	δ
0.664	0.0056	0.0056	0.0167, 0.0056	0.003, 0.0015	0.0015, 0.0003	0.2	0.0332	0.0332, 0.2238	$2.778\times 10^{-4}$

**Table A5:** Rate parameters used for Figure 11, Figure 12 and Figure 13.

$\beta_{v}$	$\gamma_{v}$	$\gamma_{P}$	μ	$\nu_{v}$	$\sigma_{P2-1}$	$\sigma_{P3-2}$	$\delta_{v}$	$\alpha_{P1-1}$	$\alpha_{P1-2}$	$\alpha_{P1-3}$
0.664	0.0028	0.0167	0.0056	0.2	0.0056	0.0056	$2.778\times 10^{-4}$	0.0491	0.0332	0.0332
$\beta_{\mathrm{uv}}$	$\gamma_{\mathrm{uv}}$	$\nu_{\mathrm{uv}}$	$\delta_{\mathrm{uv}}$	$\omega_{0}$	$\omega_{1}$	$\omega_{2}$	$\lambda_{0}$	$\lambda_{1}$	$\lambda_{2}$	
0.664	0.0056	0.2	$2.778\times 10^{-4}$	0.0045, 0.0091	0.003, 0.0045, 0.0091	0.0018, 0.0045, 0.0091	0.0015, 0.0048	0.0018, 0.0048, 0.0096	0.0009, 0.0047, 0.0094	

**Table A6:** Rate parameters used for Figure 15.

$\beta_{1}$	$\beta_{2}$	$\gamma_{1}$	$\gamma_{2}$	$\gamma_{P}$	ω	$\nu_{1}$	$\alpha_{1}$	$\alpha_{2}$	$\delta_{1}$	$\nu_{2}$	$\delta_{2}$	$\rho_{1-2}$
1.38	2	0.0056	0.0056	0.0056	0.005	0.2	0.1835	1	$2.778\times 10^{-4}$	0.2	$1.389\times 10^{-5}$	2
